# Ductility and Toughness Improvement of Injection-Molded Compostable Pieces of Polylactide by Melt Blending with Poly(ε-caprolactone) and Thermoplastic Starch

**DOI:** 10.3390/ma11112138

**Published:** 2018-10-30

**Authors:** Luis Quiles-Carrillo, Nestor Montanes, Fede Pineiro, Amparo Jorda-Vilaplana, Sergio Torres-Giner

**Affiliations:** 1 Technological Institute of Materials (ITM), Universitat Politècnica de València (UPV), Plaza Ferrándiz y Carbonell 1, 03801 Alcoy, Spain; luiquic1@epsa.upv.es (L.Q.-C.); nesmonmu@upvnet.upv.es (N.M.); fepival@epsa.upv.es (F.P.); amjorvi@upv.es (A.J.-V.); 2 Novel Materials and Nanotechnology Group, Institute of Agrochemistry and Food Technology (IATA), Spanish National Research Council (CSIC), Calle Catedrático Agustín Escardino Benlloch 7, 46980 Paterna, Spain

**Keywords:** PLA, PCL, TPS, biopolymer blends, mechanical properties, compostable plastics

## Abstract

The present study describes the preparation and characterization of binary and ternary blends based on polylactide (PLA) with poly(ε-caprolactone) (PCL) and thermoplastic starch (TPS) to develop fully compostable plastics with improved ductility and toughness. To this end, PLA was first melt-mixed in a co-rotating twin-screw extruder with up to 40 wt % of different PCL and TPS combinations and then shaped into pieces by injection molding. The mechanical, thermal, and thermomechanical properties of the resultant binary and ternary blend pieces were analyzed and related to their composition. Although the biopolymer blends were immiscible, the addition of both PCL and TPS remarkably increased the flexibility and impact strength of PLA while it slightly reduced its mechanical strength. The most balanced mechanical performance was achieved for the ternary blend pieces that combined high PCL contents with low amounts of TPS, suggesting a main phase change from PLA/TPS (comparatively rigid) to PLA/PCL (comparatively flexible). The PLA-based blends presented an “island-and-sea” morphology in which the TPS phase contributed to the fine dispersion of PCL as micro-sized spherical domains that acted as a rubber-like phase with the capacity to improve toughness. In addition, the here-prepared ternary blend pieces presented slightly higher thermal stability and lower thermomechanical stiffness than the neat PLA pieces. Finally, all biopolymer pieces fully disintegrated in a controlled compost soil after 28 days. Therefore, the inherently low ductility and toughness of PLA can be successfully improved by melt blending with PCL and TPS, resulting in compostable plastic materials with a great potential in, for instance, rigid packaging applications.

## 1. Introduction

The extensive use of petroleum-derived polymers is responsible for the increasing concern about the environmental impact of plastics due to both their origin and end-of-cycle, since most of them are not biodegradable. Worldwide polymer production was estimated to be 260 million metric tons per year in 2007 and it is considered that in 2020 each person will consume around 40 kg of plastic annually [1]. Bioplastics emerge as an alternative to conventional plastics, including both natural-sourced polymers and also petroleum-based polyesters that undergo biodegradation. Among biopolymers, polylactide (PLA) is currently considered one of the most promising biopolyester at industrial scale due to its good balance between physicochemical properties, low price, and sustainability [2]. PLA is obtained from lactide derived from starch fermentation and it is fully biodegradable. The increasing use of PLA in the last years is noticeable with a current worldwide production of about 140,000 tons/year [3]. The main uses of PLA cover a wide variety of industrial sectors for instance automotive [4,5,6], biomedical applications [7,8], packaging [9,10] or, lately, the growing industry of 3D printing [11,12]. Despite this, PLA presents several intrinsic restrictions that are mainly related to its relatively high price, low heat resistance, and high fragility [13]. As a result, PLA cannot fulfill the technical requirements of some industries, limiting its expansion to commodity areas such as food packaging [14].

To overcome or, at least, minimize the low ductility and toughness of PLA, several approaches have been considered with excellent results. The first approach is copolymerization. For instance, the simultaneous polymerization of lactide acid (LA) with glycolic acid (GA) leads to the synthesis of poly(lactic acid-*co*-glycolic acid) (PLGA). In general terms, PLGA copolymers exhibit improved solubility as well as better ductile properties than both PLA and poly(glycolic acid) (PGA) homopolymers [15,16]. Nevertheless, copolymers are frequently expensive and their use is not yet generalized at industrial scale. A second strategy to increase PLA toughness is focused on the use of plasticizers. Some of the widely used plasticizers for PLA include poly(ethylene glycol) (PEG) [17], triethyl citrate (TEC) [18,19], and oligomers of lactic acid (OLAs) [20]. All these plasticizers contribute positively to increasing ductility by providing a relevant decrease in the glass transition temperature (T_g_) of PLA but they can also reduce the heat resistance, tensile strength, and stiffness. In addition to these plasticizers, in recent years, new vegetable oil-derived plasticizers have been successfully developed for PLA formulations such as maleinized, acrylated, hydroxylated, and epoxidized vegetable oils [21,22,23,24]. Although their efficiency as primary plasticizers for PLA is lower than those indicated previously, the particular chemical structure of these multi-functionalized modified vegetable oils delivers chain extension, branching and, in some cases, cross-linking resulting in improved toughness without compromising the mechanical strength in a great extent [23]. The third route is related to the manufacturing of PLA-based blends. This represents a very cost-effective solution to reduce the intrinsic fragility of PLA materials without significantly decreasing their tensile strength. A wide variety of binary blends based on PLA has been extensively studied in the last years. For instance, it is worthy to note the interest in binary blends of PLA with polyhydroxyalkanoates (PHAs) [25,26], polyamides (PAs) [27,28], poly(butylene adipate-*co*-terephthalate) (PBAT) [29,30], thermoplastic starch (TPS) [31], poly(ε-caprolactone) (PCL), poly(butylene succinate) (PBS), and poly(butylene succinate-*co*-adipate) (PBSA) [32,33,34]. These previous studies are based on the fact that, to improve toughness, PLA is blended with flexible polymers that perform as a rubber-like phase inside a rigid polymer matrix as, for instance, polybutadiene rubbers (BRs) do in high-impact polystyrene (HIPS).

In addition to binary blends, a wide variety of ternary blends based on PLA have been proposed to tailor the desired properties, particularly in terms of improved toughness [35,36,37]. On the one hand, PCL is a well-known synthetic aliphatic biopolyester, characterized by a high crystallinity, relatively fast biodegradability, and high ductility. However, PCL shows a low melting temperature (T_m_), of about 60 °C, which restricts its use in a wide range of applications [38]. PLA/PCL blends are attracting some industrial uses since flexible PCL domains can be finely dispersed into the rigid PLA matrix leading to improved toughness without compromising biodegradation [39]. In addition, the resultant blends are fully resorbable, finding interesting applications as biomedical devices. On the other hand, starch is a versatile and useful biopolymer. Starch has to be modified by means of plasticizers (e.g., glycerol and water) [40] and/or chemical reaction (e.g., esterification) [41] in order to be melt-processed, which then results in TPS. The role of plasticizers is to destructurize granular starch by breaking hydrogen bonds between the starch macromolecules, accompanying with a partial depolymerization of starch backbone. As a result, TPS leads to compostable plastic materials offering interesting opportunities in the packaging field due to its low cost and tailor-made mechanical behavior by selecting the appropriate plasticizers [42]. Blending of PLA with TPS is, therefore, a good way to balance the price and develop materials that has new performances.

The aim of this work was to prepare and characterize ternary blends of PLA with PCL and TPS to overcome the intrinsic brittleness of PLA. To this end, different PCL and TPS contents were blended by melt compounding with PLA to obtain PLA-based materials with tailor-made properties. The resultant PLA/PCL/TPS ternary blends were, thereafter, injection-molded into pieces and subjected to mechanical, morphological, thermal, and thermomechanical analysis while their potential compostability was also ascertained.

## 2. Materials and Methods

### 2.1. Materials

Commercial PLA Ingeo™ biopolymer 6201D was purchased from NatureWorks (Minnetonka, MN, USA). This PLA resin has a density of 1.24 g·cm^−3^, a met flow rate (MFR) of 15–30 g·10 min^−1^ (210 °C, 2.16 kg), a T_g_ value in the 55–60 °C range, and a T_m_ value in the 165–175 °C range. This MFR allows the manufacturing of PLA articles by both extrusion and injection molding. PCL was a Capa^TM^ 6800 commercial grade supplied by Perstorp UK Ltd. (Warrington, UK) with a density of 1.15 g·cm^−3^, a T_g_ value of −60 °C, and a T_m_ value in the 58–62 °C range. The melt flow index (MFI) of PCL is 2–4 g·10 min^−1^ (160 °C, 2.16 kg). TPS Mater-Bi^®^ NF 866 was obtained from Novamont SPA (Novara, Italy), which is derived from maize starch. Its MFI is 3.5 g·10 min^−1^ (150 °C, 2.16 kg). This TPS resin presents a density of 1.27 g·cm^−3^, a T_g_ value ranging from −35 °C to −40 °C, and a T_m_ value in the 110–120 °C range.

### 2.2. Manufacturing of Ternary PLA/PCL/TPS Blends

Prior to manufacturing, all the biopolymer pellets were dried at 45 °C for 48 h in a MDEO dehumidifier from Industrial Marsé (Barcelona, Spain). All blends contained 60 wt % PLA while PCL and TPS varied from 0 to 40 wt % to give a series of materials with different properties. The corresponding amounts of each biopolymer is summarized and coded in Table 1. 

Initially, the biopolymer pellets were weighed and manually mixed in a zipper bag. Then, the different mixtures were melt-compounded in a co-rotating twin-screw extruder from Construcciones Mecánicas Dupra S.L. (Alicante, Spain) at a rotating speed of 30 rpm. The screws had a diameter of 25 mm with a length-to-diameter ratio (L/D) of 24. The temperature profile, from the feeding hopper to the extrusion die (circular), was set at 165 °C–170 °C–175 °C–180 °C. The extruded materials were pelletized in an air-knife unit.

The compounded pellets were finally processed by injection molding in a Meteor 270/75 injection machine from Mateu and Solé (Barcelona, Spain). The temperature profile during the injection molding process was: 160 °C (hopper)–165 °C–170 °C–180 °C (injection nozzle). A clamping force of 75 tons was applied while the cavity filling and cooling time were set at 1 and 10 s, respectively. Pieces with a mean thickness of 4 mm were produced.

### 2.3. Mechanical Characterization

Tensile and flexural tests were performed on the injection-molded pieces of PLA and its blends using a universal test machine ELIB 50 from S.A.E. Ibertest (Madrid, Spain). Tensile tests were carried out following the guidelines of ISO 527-1:2012 using a cross-head speed rate of 10 mm·min^−1^. Similarly, flexural tests were carried out according to ISO 178 and the speed rate was 5 mm·min^−1^. Both tests were carried out at 25 °C and with a load cell of 5 kN. At least six samples of each material were tested.

Shore D hardness of the biopolymer pieces were obtained in a Shore durometer 676-D from J. Bot Instruments (Barcelona, Spain), as recommended by ISO 868:2003. A type-D indenter with a load of 5 kg and an indentation time of 12–15 s was used to stabilize the measurement. The impact-absorbed energy, which is directly related to toughness, was estimated by using the Charpy impact test with a 1-J pendulum from Metrotec S.A. (San Sebastian, Spain). The average energy per unit cross-section area was obtained on V-notched samples with a radius of 0.25 mm, as recommended by ISO 179-1:2010. Both mechanical tests were carried out at room temperature, that is, 25 °C, and five different samples of each material were tested.

### 2.4. Morphological Characterization

The morphology of the fracture surfaces was studied on the broken samples after the impact tests by field emission scanning electron microscopy (FESEM) in a ZEISS ULTRA 55 microscope from Oxford Instruments (Abingdon, UK). Before placing the samples into the vacuum chamber, all surfaces were covered with a thin metallic layer of gold-palladium by sputtering in an EMITECH mod. SC7620 from Quorum Technologies, Ltd. (East Sussex, UK). The acceleration voltage for the FESEM study was 2 kV.

### 2.5. Solubility

The relative affinity of the biopolymers was estimated by measuring the solubility parameters (δ) according to the Small’s method [43]. To consider the blend miscible, the δ values of the polymers should be of the same order. This parameter was determined according to Equation (1):

(1) $$\delta =\frac{\rho \cdot \Sigma G}{M_{n}},$$

where ρ is the density of the polymer, M_n_ is the molar mass of the repeating unit, and ΣG is the sum of the group contributions to the cohesive energy density.

### 2.6. Thermal Characterization

The thermal transitions of PLA and its blends were obtained by differential scanning calorimetry (DSC) in a Mettler-Toledo 821 calorimeter (Schwerzenbach, Switzerland). An average sample weight comprised in the 5–7 mg range was used for all DSC tests. The thermal program consisted of a first heating step from 25 °C to 190 °C, followed by a cooling step down to 25 °C, and a second heating step up to 300 °C. All heating rates were set at 10 °C·min^−1^. A constant nitrogen flow-rate of 66 mL·min^−1^ was used to achieve inert atmosphere. Aluminum pans with a total volume capacity of 40 µL were used.

Thermal stability was determined by thermogravimetric analysis (TGA) in a Mettler-Toledo TGA/SDTA 851 thermobalance (Schwerzenbach, Switzerland). Samples with an average size of 5–7 mg were placed into standard alumina crucibles with a total volume capacity of 70 µL and subjected to a heating program from 30 °C to 650 °C at a heating rate of 20 °C·min^−1^ in air atmosphere.

### 2.7. Thermomechanical Characterization

The effect of temperature on the mechanical properties was followed by dynamic mechanical thermal analysis (DMTA) in an oscillatory rheometer AR-G2 from TA Instruments (New Castle, DE, USA). This rheometer is equipped with a special clamp system to work with solid samples in a combined torsion/shear mode. Injection-molded pieces with dimensions of 4 mm × 10 mm × 40 mm were subjected to a temperature sweep from −80 °C to 120 °C at a constant heating rate of 2 °C·min^−1^. The selected frequency was 1 Hz and the maximum shear deformation was set at 0.1% (% γ).

The thermomechanical behavior of the ternary blends was also assessed by obtaining the Vicat softening temperature (VST) and the heat deflection temperature (HDT) in a Vicat/HDT station VHDT 20 from Metrotec S.A. (San Sebastián, Spain). VST was obtained following the procedure described in ISO 306, using the B50 heating method and applying a total force of 50 N at a heating rate of 50 °C·h^−1^. Regarding HDT, ISO 75-1 recommendations were followed. To this end, samples sizing 4 mm × 10 mm × 80 mm were placed between two supports with a total span of 60 mm. After this, a load of 320 g was applied in the center using a heating rate of 120 °C·h^−1^.

### 2.8. Disintegration Test

A disintegration test in controlled compost conditions was conducted following the guidelines of ISO 20200 at a temperature of 58 °C and a relative humidity (RH) of 55%. For this, squared thermo-compressed samples sizing 1 mm × 30 mm × 30 mm were placed in a carrier bag and buried in a controlled soil with the following composition (in dry weight): sawdust (40 wt %), rabbit-feed (30 wt %), ripe compost (10 wt %), corn starch (10 wt %), saccharose (5 wt %), corn seed oil (4 wt %), and urea (1 wt %). To follow the disintegration process, samples were periodically unburied, washed with distilled water, dried, and weighed in an analytic balance. In order to get a visual evolution of this process, pictures of the disintegration process were also collected. The weight loss due to disintegration in the controlled compost soil was calculated by means of Equation (2):

(2) $$\mathrm{Weight}\;\mathrm{loss}\left(\%\right)=\left(\frac{W_{0}-W_{t}}{W_{0}}\right)\cdot 100,$$

where W_t_ is the weight of the sample after a bury time t and W_0_ is the initial dry weight of the sample. All tests were carried out in triplicate to ensure reliability.

### 2.9. Statistical Analysis

Ternary graphs were plotted using Origin Pro 2015 from OriginLab Corporation (Northampton, MA, USA) with the Ternary Contour function using the average and standard deviation values.

## 3. Results

### 3.1. Mechanical Properties

**Figure 1:**
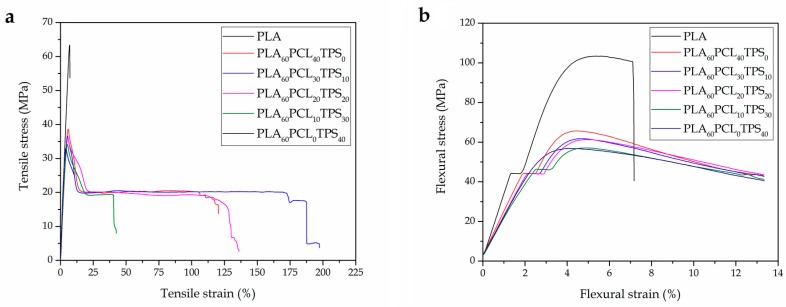
Stress–strain curves of the polylactide (PLA), poly(ε-caprolactone) (PCL), and thermoplastic starch (TPS) blend pieces obtained from: (**a**) tensile test; and (**b**) flexural test.

The injection-molded pieces of PLA and of the binary and ternary blends of PLA with PCL and TPS were tested in order to determine their mechanical properties. The tensile strength (σ_tensile_) and elongation at break (ε_b_) were obtained under tensile conditions, while the flexural modulus (E_flexural_) and flexural strength (σ_flexural_) were determined under flexural conditions. Figure 1 shows the resultant stress–strain curves of the injection-molded PLA-based pieces obtained during the tensile tests (Figure 1a) and flexural tests (Figure 1b). 

**Figure 2:**
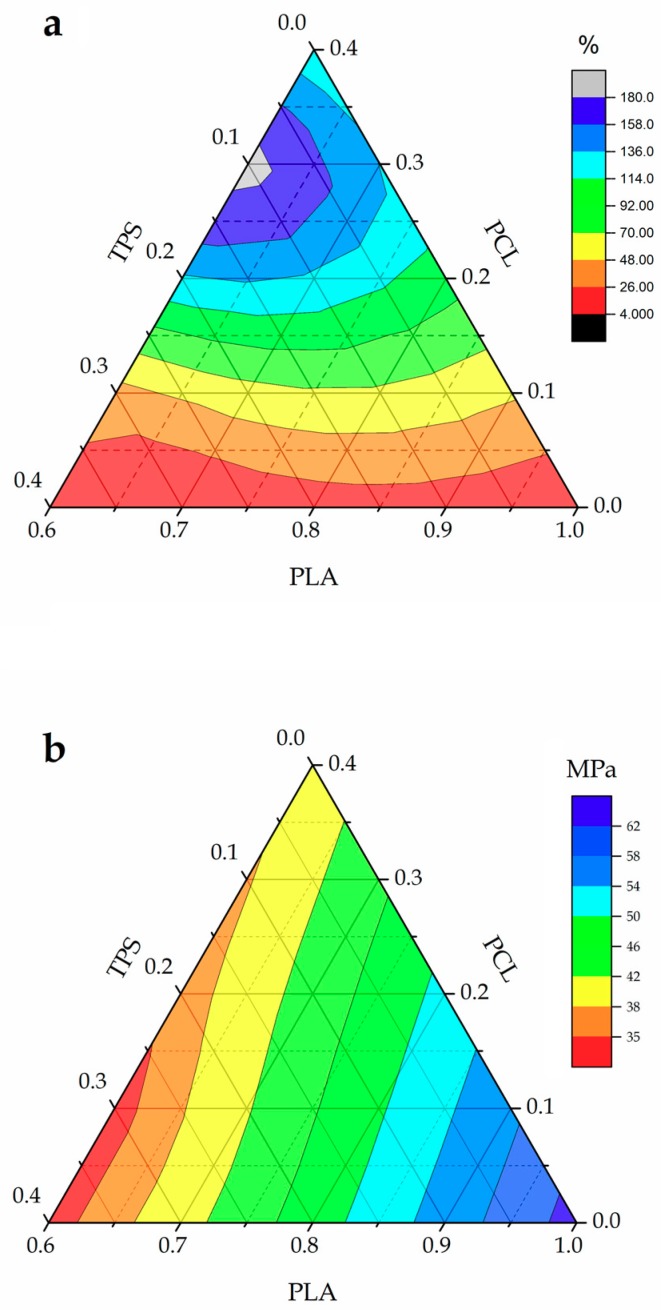
Ternary graphs showing the evolution of the mechanical properties of the polylactide (PLA), poly(ε-caprolactone) (PCL), and thermoplastic starch (TPS) blend pieces in terms of: (**a**) elongation at break (ε_b_); and (**b**) tensile strength (σ_tensile_).

Figure 2 summarizes in ternary graphs the evolution of the tensile properties, that is, ε_b_ and σ_tensile_, of the injection-molded PLA-based pieces with the addition of PCL and TPS. One can observe in Figure 2a that the neat PLA piece was very fragile, presenting a ε_b_ value of 4.9%. This value, together with a medium-to-high σ_tensile_ value of 63.4 MPa, was responsible for its high brittleness. As one can see, the addition of both PCL and TPS provided a positive effect on the PLA’s ductility, but this effect was much more pronounced with PCL due to its intrinsic higher flexibility compared to TPS. In particular, the PLA_60_PCL_30_TPS_10_ and PLA_60_PCL_20_TPS_20_ blend pieces showed a remarkable increase in ε_b_ with values of 196.7% and 134.3%, respectively, which were noticeably higher than that of the neat PLA piece. It is also worthy to note that these two ternary blend pieces presented higher ductility than the binary blend piece of PLA with PCL, that is, PLA_60_PCL_40_TPS_0_, which suggests a synergistic effect of both PCL and TPS on the overall material’s ductility. With regard to the mechanical strength of the PLA-based pieces, as shown in Figure 2b, one can observe that the pieces presented lower σ_tensile_ values after the addition of PCL and TPS. In the case of the binary blend piece of PLA with PCL, that is, PLA_60_PCL_40_TPS_0_, the value of σ_tensile_ was reduced to 39.1 MPa, which is remarkable lower than that observed for the neat PLA piece. The binary blend piece of PLA with TPS, that is, PLA_60_PCL_0_TPS_40_, resulted in even a lower σ_tensile_ value, that is, 33.6 MPa. All intermediate compositions showed a proportional decrease depending on the PCL and TPS content. With regard to the blend pieces containing 30 wt % and 40 wt % TPS, that is, PLA_60_PCL_10_TPS_30_ and PLA_60_PCL_0_TPS_40_, respectively, the ductility was poor when compared to the ternary blend piece with the highest PCL content, that is, PLA_60_PCL_30_TPS_10_. This suggests that both individual PCL and TPS biopolymers have a positive effect on the ductile properties of PLA but the best results were obtained for the ternary blends that combined a high PCL content with low amounts of TPS. The addition of 40 wt % TPS to PLA without PCL, that is, PLA_60_PCL_0_TPS_40_, produced the piece with the poorest mechanical performance. Although this piece doubled the ductility of the neat PLA piece, that is, ε_b_ increased to 8.8%, σ_tensile_ also decreased to a value of 33.6 MPa. As previously indicated, the binary blend piece made of PLA with 40 wt % PCL, that is, PLA_60_PCL_40_TPS_0_, also provided non-optimum results showing a value of ε_b_ of 114.3%. However, interestingly, the ternary blend pieces containing 20–30 wt % PCL and 20–10 wt % TPS, that is, PLA_60_PCL_20_TPS_20_ and PLA_60_PCL_30_TPS_10_, offered the best ductile properties with remarkable high ε_b_ values. 

The above-described observation suggests that a main phase change, from PLA/TPS (comparatively rigid) to PLA/PCL (comparatively flexible), occurred in the ternary blends when relative high contents of PCL and low contents of TPS are blended with PLA. In this sense, other authors have reported that the ductility of PLA/TPS blends can be drastically increased by the incorporation of high amounts of flexible polyesters. For instance, Zhen et al. [44] observed that the addition of PBS led to a mechanical strength decrease and ductility increase in TPS/PLA blends. Whereas the σ_tensile_ values decreased from 28.54 MPa to 14.60 MPa with the increase of PBS content from 0% to 50 wt %, the values of ε_b_ of the ternary blends also increased from 1.82% to 45.17%. However, the most significant mechanical changes were obtained for PBS contents above 20 wt %, which was ascribed to the main phase change from TPS/PLA (comparatively rigid) to TPS/PBS (comparatively flexible). Similar results were previously obtained by Ren et al. [45] for ternary TPS/PLA/PBAT blends, in which the main phase changed from TPS/PLA (comparatively rigid) to TPS/PBAT (comparatively flexible) when the PBAT reached contents between 20 and 30 wt %.

**Figure 3:**
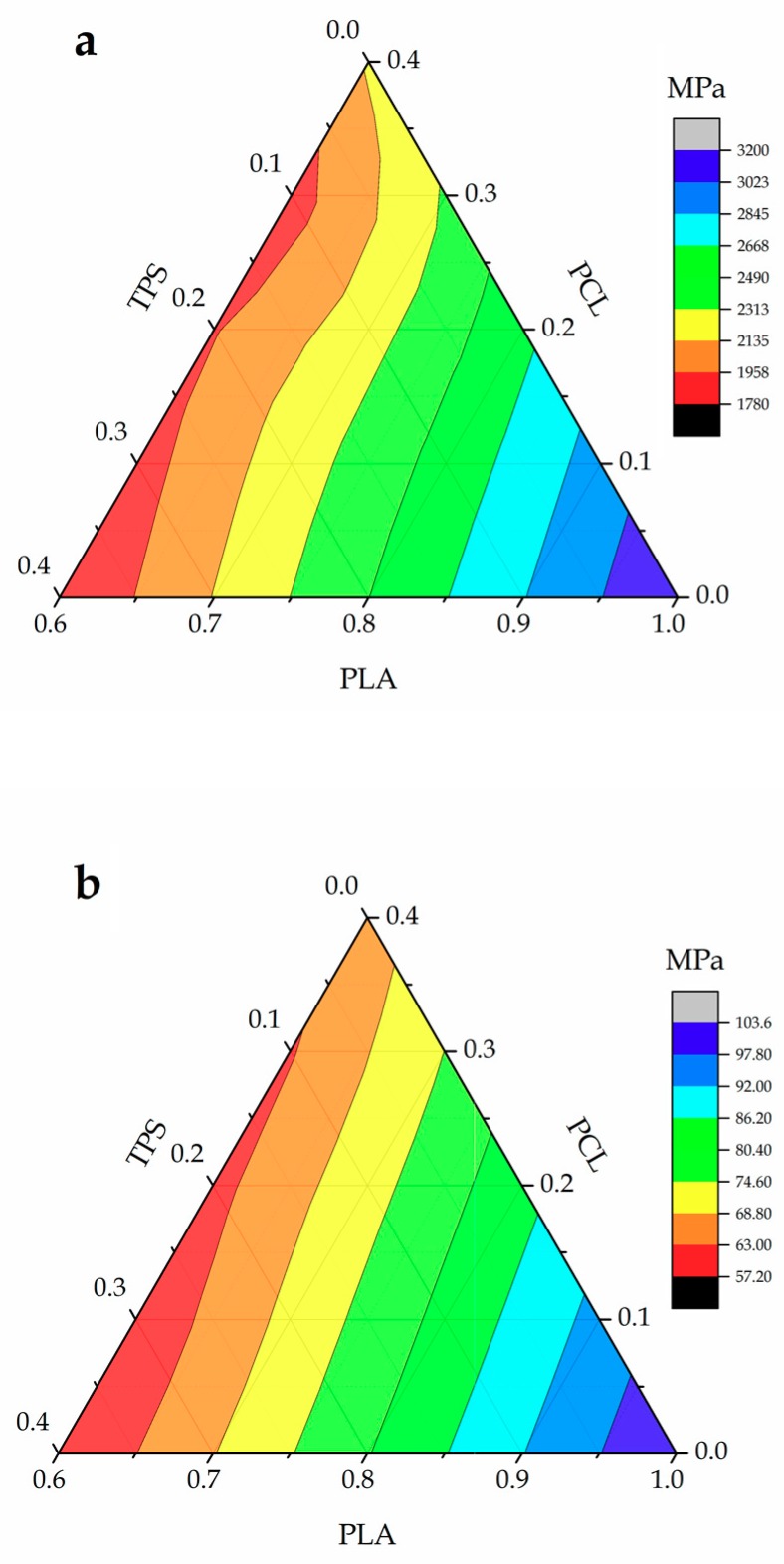
Ternary graphs showing the evolution of the mechanical properties of the polylactide (PLA), poly(ε-caprolactone) (PCL), and thermoplastic starch (TPS) blend pieces in terms of: (**a**) flexural modulus (E_flexural_); and (**b**) flexural strength (σ_flexural_).

Figure 3 shows a ternary graph with the evolution of flexural properties of the injection-molded PLA-based pieces, that is, E_flexural_ and σ_flexural_, when varying the composition of the blends. With regard to E_flexural_, in Figure 3a it can be seen that a clear reduction was observed after the incorporation of PCL and/or TPS in comparison to the neat PLA piece. In fact, it was reduced from 3200 MPa, for the neat PLA piece, to 2100 MPa, for the binary blend piece of PLA with 40 wt % PCL, that is, PLA_60_PCL_40_TPS_0_. The value of E_flexural_ followed the same tendency as reported by Ferry et al. [46], decreasing as the TPS content increased in PLA/TPS blends. In particular, E_flexural_ presented the lowest value, that is, 1780 MPa, for the binary blend piece of PLA with 40 wt % TPS, that is, PLA_60_PCL_0_TPS_40_. As shown in Figure 3b, σ_flexural_ decreased from 103 MPa, for the neat PLA piece, down to values of 65 MPa and 57 MPa for the binary blend pieces containing 40 wt % PCL, that is, PLA_60_PCL_40_TPS_0_, and 40 wt % TPS, that is, PLA_60_PCL_0_TPS_40_, respectively. Intermediate compositions of the ternary blends showed a proportional decrease in the σ_flexural_ values as a function of their composition. Similar results were reported by García-Campo et al. [47] where intermediate compositions of the ternary PLA/PHB/PCL blends presented an intermediate mechanical behavior between the binary PLA/PHB and PLA/PCL blends.

As stated above, one of the main drawbacks of PLA is its low toughness. Table 2 summarizes the main results obtained from the Charpy impact test as well as the Shore D hardness measurements. As it can be observed, the typical energy absorption of the V-notched neat PLA piece was very low, of about 2.14 kJ·m^−2^. With regard to the binary blend piece with 40 wt % PCL, that is, PLA_60_PCL_40_TPS_0_, it resulted in an impact energy per unit cross-section of 6.52 kJ·m^−2^, which represents an increase of more than three times compared to the neat PLA piece. Similar findings were reported for instance by Chen et al. [48], showing a remarkable improvement in the PLA toughness by the addition of PCL. The ternary blend pieces with 10–30 wt % PCL also showed relatively high values of impact strength, thus, supporting the good effect of PCL on the overall PLA toughness. It is also important to remark that the binary blend piece of PLA with 40 wt % TPS, that is, PLA_60_PCL_0_TPS_40_, provided increased toughness with an impact strength value of 5.46 kJ·m^−2^. However, as observed above for other mechanical properties, the effect of PCL was more intense than that of TPS. In relation to the Shore D hardness, the hardness value of the neat PLA piece was 73.1. The Shore D hardness values decreased by approximately 10 units in all the developed blend pieces, thus, reaching a plateau at values of 63–64.

### 3.2. Morphology

**Figure 4:**
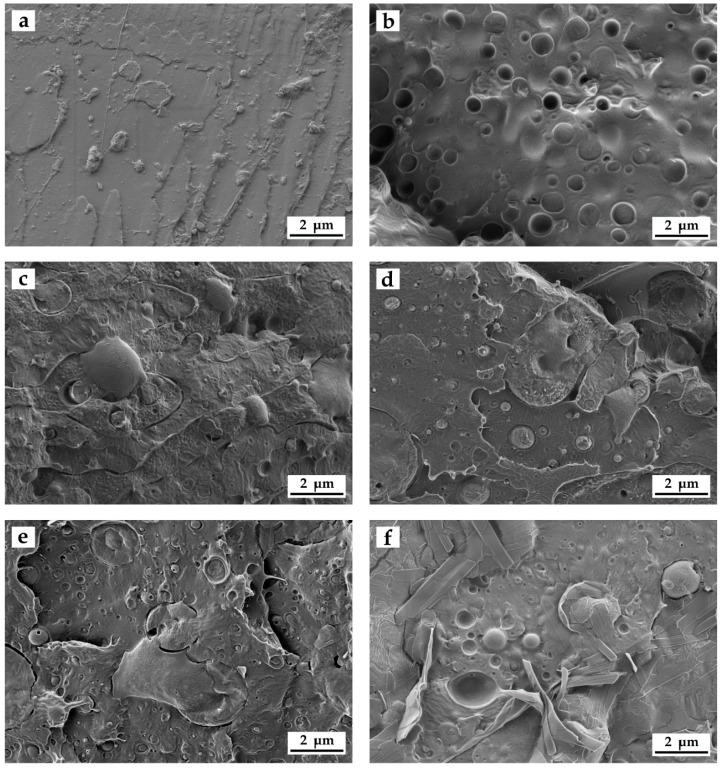
Field emission scanning electron microscopy (FESEM) images of the fracture surfaces of the polylactide (PLA), poly(ε-caprolactone) (PCL), and thermoplastic starch (TPS) blend pieces: (**a**) Neat PLA; (**b**) PLA_60_PCL_40_TPS_0_; (**c**) PLA_60_PCL_30_TPS_10_; (**d**) PLA_60_PCL_20_TPS_20_; (**e**) PLA_60_PCL_10_TPS_30_; and (**f**) PLA_60_PCL_0_TPS_40_. Images were taken at 5000× and scale markers are of 2 µm.

Figure 4 shows the FESEM images corresponding to fracture surfaces of the different injection-molded PLA-based pieces obtained after the impact tests. Figure 4a, which corresponds to the neat PLA piece, shows the typical fracture surface of a brittle material with low roughness, that is, a smooth and relatively flat surface. Regarding the binary blend piece of PLA with 40 wt % PCL, that is, PLA_60_PCL_40_TPS_0_, shown in Figure 4b, a clearly different fracture surface can be observed. In particular, the surface roughness was higher and the flat surface changed to an “island-and-sea” morphology that was based on finely dispersed PCL-rich domains, sizing 1–5 µm, into the PLA matrix. Although PLA and PCL are thermodynamically immiscible [49], this particular structure positively contributed to improving toughness as the enclosed microdroplets of PCL were able to absorb energy, acting as a rubber-like phase dispersed in a brittle matrix [50]. Plastic deformation provided by PCL can be also observed by the presence of some filaments along the PLA matrix. Addition of 10 wt % TPS in the ternary blend piece, that is, PLA_60_PCL_30_TPS_10_, also produced a noticeable change in the morphology, which can be observed in Figure 4c. In particular, one can observe that the TPS-rich domains presented a higher size, in the 1–35 µm range. A similar morphology was previously reported by Sarazin et al. [31]. In Figure 4d–f one can observe that, as the TPS content increased, the TPS-rich domains increased both in number and size, which is an indication of their poor interfacial interaction with the PLA-based matrix [51]. With regard to the blend pieces with the highest TPS contents, that is, both PLA_60_PCL_10_TPS_30_ and PLA_60_PCL_0_TPS_40_, the domains changed from spherical to a ribbon-like morphology due to stretching of the TPS phase during fracture. This morphological changes were also observed by Carmona et al. [52] in TPS/PCL/PLA blends at high TPS contents, that is, 33.3 wt % TPS. Ferri et al. [49] have previously related the formation of TPS flakes to the crystalline plane growth or “crystalline lamellae” located at the amylopectin branches that fold up during fracture. In particular, the mechanically-induced flakes structures form parallel-plane blocks and clusters, resulting in granules separated by porous of amorphous areas in which amylose and plasticizers can be allocated. Since PLA is a hydrophobic biopolymer whilst TPS is highly hydrophilic, indeed one of the main drawbacks of TPS is related to its extremely high moisture sensitiveness, this results in the lack (or very low) affinity between the two biopolymers that frequently leads to a strong phase separation [53].

To further study the compatibility of the developed blends and also to ascertain their resultant morphologies, the miscibility of the biopolymer formulations was evaluated using the Small’s method. According to this, the closer the δ values, the higher the miscibility of the polymers in the blend. Table 3 shows the chemical structure and the resultant δ values of the here-studied biopolymers. One can observe that both PLA and PCL presented a relatively similar δ value while TPS presented a considerably lower value, which support the above-described mechanical and morphological results. This difference in the δ values can be mainly related to the higher density of oxygen atoms in the chemical structure of TPS, mainly hydroxyl groups (–OH), which are certainly responsible for its high hydrophilicity. However, it is also worthy to mention that the δ values obtained for TPS can also vary considerably due to the thermoplastic carbohydrate is obtained by mixing with large quantities of plasticizers. The here-reported δ values are in agreement with Samper et al. [54] who obtained values for PLA and TPS of 19.1–20.1 and 8.4, respectively. Similarly, Bordes et al. [55] reported a δ value of 17 MPa^1/2^ for PCL. 

### 3.3. Thermal Properties

**Figure 5:**
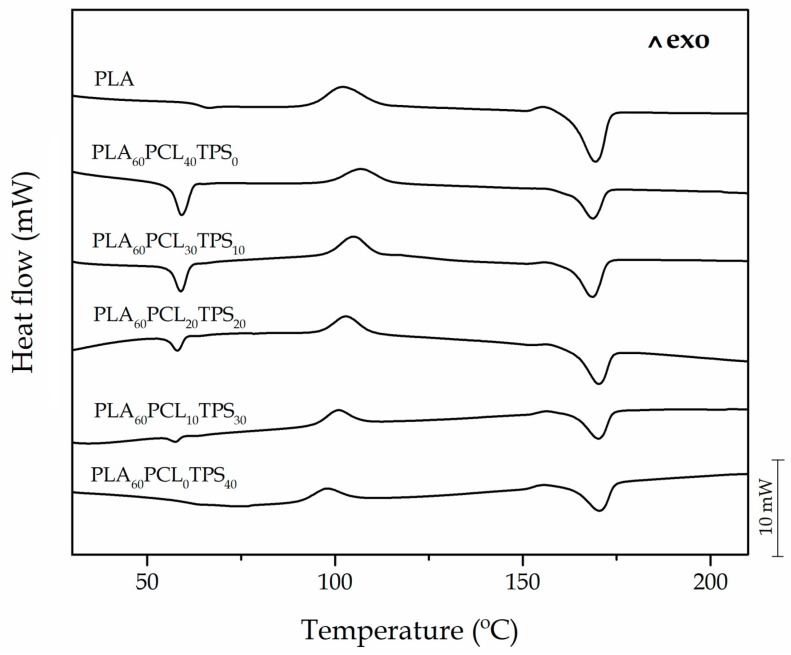
Comparative plot of the differential scanning calorimetry (DSC) curves of the polylactide (PLA), poly(ε-caprolactone) (PCL), and thermoplastic starch (TPS) blend pieces.

Figure 5 shows a comparison plot of the DSC curves obtained during the second heating cycle performed on the injection-molded PLA-based pieces. One can observe that the neat PLA piece showed a T_m_ value of 169.5 °C. In addition, PLA developed cold crystallization with a cold crystallization temperature (T_CC_) located at approximately 103 °C and a value of T_g_ of around 63 °C. In the DSC curve for the binary blend piece of PLA with 40 wt % PCL, that is, PLA_60_PCL_40_TPS_0_, it can be observed that the melt peak intensity for PLA was lower due to the diluting effect of PCL. An additional melting process with a peak located at ~59 °C appeared, which is attributable to the PCL’s T_m_. This melting process overlapped with the glass transition region of PLA so that it was not possible to separate both processes by conventional DSC. Similar results were also obtained by, for instance, Navarro-Baena et al. [57] for PLA/PCL blends using dynamic DSC measurements. In addition, the value of T_m_ for PLA did not remarkably change in the blends. As the PCL content in the ternary blends decreased, the corresponding peak intensity, that is, the melting enthalpy (ΔH_m_), also decreased. In the case of the blend piece of PLA with 40 wt % TPS, that is, PLA_60_PCL_0_TPS_40_, it also showed a slight shift of the cold crystallization region towards lower temperatures, which can be ascribed to a plasticizing effect of the PLA matrix by TPS. In this sense, it is worthy to note that TPS contains high amounts of plasticizers, such as glycerol, which can contribute to plasticizing PLA. The resultant plasticization is also evident by observing the PLA’s T_g_, which moved down to 61.2 °C. The glass transition regions of both PCL and TPS were not registered using the present thermal program since these peaks are located below room temperature, in particular from −50 °C to −65 °C for PCL [42] and from −75 °C to 10 °C for TPS [58,59].

**Figure 6:**
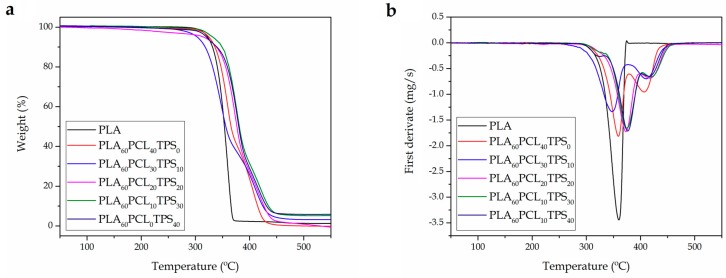
Comparative plot of the polylactide (PLA), poly(ε-caprolactone) (PCL), and thermoplastic starch (TPS) blend pieces in terms of: (**a**) Thermogravimetric analysis (TGA) curves; and (**b**) first derivative thermogravimetric (DTG) curves.

Figure 6 gathers the TGA thermograms (Figure 6a) and their corresponding first derivative thermogravimetric (DTG) curves (Figure 6b) of the injection-molded PLA-based pieces. Additionally, Table 4 summarizes the main thermal values obtained from these curves. It can be observed that the PLA_60_PCL_30_TPS_10_ piece showed the lowest thermal stability, having the decomposition process in two stages. Its typical thermal degradation parameters, that is, the onset degradation temperature (T_5%_) and degradation temperature (T_deg_), were 303.5 °C and 348 °C, respectively. Regarding the neat PLA piece, although it showed a high T_5%_ value, that is, 322 °C, its degradation occurred in a single step at a relatively low T_deg_ value, that is, 360 °C. In contrast, the PLA_60_PCL_30_TPS_10_ piece and, in particular, the PLA_60_PCL_10_TPS_30_ piece, improved the thermal stability by having lower mass losses at high temperatures while their T_deg_ values showed an increase of up to 15 °C with regard to the neat PLA. Therefore, the addition of both PCL and TPS led to an increase of the thermal stability of PLA at high temperatures. In addition, the binary and ternary pieces presented a thermal degradation process in two steps. The first mass loss corresponds to the PLA degradation while the second, at higher temperatures, can be attributed to the PCL and TPS decompositions. Additionally, the PLA degradation onset was delayed by the presence of both PCL and TPS. In this sense, Patrício et al. [60] reported that the addition of PCL can successfully enhance the thermal stability of PLA. In particular, it was observed an increase in the T_deg_ value from 325 °C, for the neat PLA, up to 334 °C, for binary blends of PLA with PCL at different ratios. Mofokeng et al. [61] however suggested the lack of miscibility between PLA and PCL, indicating completely independent degradation stages for each biopolymer phase in the blend.

With regard to the residual mass, it can be observed that TPS contributed to generating a higher amount of residue. Whereas the neat PLA piece resulted in a very low char content, of approximately 1.5%, this value increased up to 6.4% in the binary blend of PLA with 40 wt % TPS, that is, PLA_60_PCL_0_TPS_40_. Thus, intermediate compositions led to intermediate char residues. This result can be related to additives incorporated into the biopolymer by the manufacturer.

### 3.4. Thermomechanical Properties 

**Figure 7:**
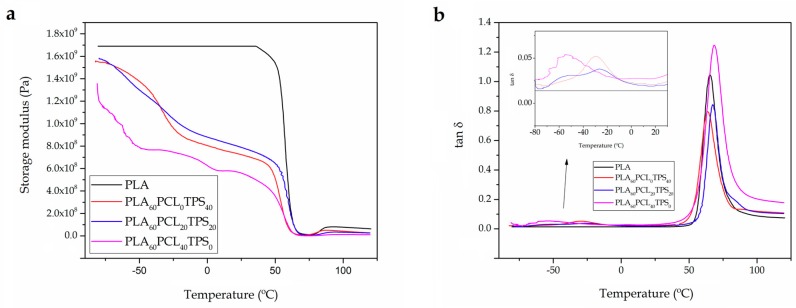
Comparative plot of the polylactide (PLA), poly(ε-caprolactone) (PCL), and thermoplastic starch (TPS) blend pieces in terms of: (**a**) Storage modulus (G’) versus temperature; and (**b**) dynamic damping factor (tan δ) versus temperature.

DMTA allows estimating the effect of temperature on the mechanical performance. Additionally, it is a more sensitive technique to evaluate potential changes in T_g_ that, in turn, can be directly related to miscibility in polymer blends [62]. Figure 7 shows the evolution of the storage modulus (G’) and the dynamic damping factor (tan δ) as a function of temperature in the injection-molded PLA-based pieces. Figure 7a presents the G’ curves for the neat PLA piece and for the binary and ternary blend pieces of PLA with PCL and TPS. G’ is directly related to the stored elastic energy and, consequently, can be directly related to stiffness. Regarding the neat PLA piece, its G’ value was 1.69 GPa at −80 °C. One can also observe that the G’ value increased from 5.6 MPa, at 80 °C, to 76 MPa, above 90 °C. This stiffness increase is ascribed to the cold crystallization process of PLA due to the rearrangement of the biopolyester chains to give a more packed structure [63]. Addition of 40 wt % TPS led to lower G’ values. For instance, the PLA_60_PCL_0_TPS_40_ piece presented a G’ value of 1.55 GPa at −80 °C and the same trend that in the case of PLA was observed at higher temperatures. The highest decrease in G’ was obtained for the binary blend piece of PLA with 40 wt % PCL, that is, PLA_60_PCL_40_TPS_0_, with a value of 1.30 GPa at −80 °C. Therefore, the addition of both PCL and TPS represents an interesting strategy to obtain PLA-based toughened formulations. In relation to the intermediate compositions, for instance the PLA_60_PCL_20_TPS_20_ piece, it is worthy to note the remarkable decrease in G’ observed at about −60 °C, which corresponds to the glass transition of PCL. Another important decrease in G’ was observed in the ternary blend pieces in the thermal region located from −20 °C to −30 °C, which is attributable to the glass transition of TPS.

Figure 7b shows the evolution of tan δ that is, the ratio of G’’ to G’, versus temperature. The alpha (α)-relaxation regions of each biopolymer can be clearly identified by the peaks of the tan δ plots, which are related to their T_g_s and molecular motions [64]. One can observe that the α-peak of the PLA phase slightly changed in the pieces when it was melt blended with the other biopolymers. In particular, it increased from 65.2 °C, for the neat PLA piece, to 68.4 °C, for the binary blend piece containing 40 wt % PCL, that is, PLA_60_PCL_40_TPS_0_, while it was reduced to 64.0 °C, for the binary blend piece containing 40 wt % TPS, that is, PLA_60_PCL_0_TPS_40_. In this sense, Martin et al. [65] observed that the α-relaxation region of the PLA phase presented a gradual decrease with increasing the amounts of TPS. In particular, the T_g_ of PLA decreased from 67 °C, for neat PLA, to about 55 °C, for PLA blends containing 10 wt % TPS. Since it was observed that glycerol has a relatively low effect on the glass transition of PLA, the shift of the α-relaxation to lower temperatures suggested some interaction between TPS and PLA and, as a result, partial miscibility between the two biopolymers was inferred. However, since this reduction was moderate, a small degree of miscibility between the blend components was concluded. In relation PCL, Mittal et al. [66] showed that the α-relaxation region of PLA occurred at higher temperatures as the amount of PCL in the binary blends was increased. In particular, the α-peak of the neat PLA increased from approximately 55 °C to 61 °C. This effect was ascribed by the authors to a better intermixing of the phases in the presence of PCL. Additionally, one can also observe that the α-peak values for the PCL- and TPS-rich phases in the blend pieces were located at approximately −55 °C and −30 °C, respectively.

Furthermore, one can observe in Table 5 that the addition of both PCL and TPS yielded lower VST and HDT values than those observed for the neat PLA piece. These results are in agreement with the above-described mechanical and thermomechanical results due to both PCL and TPS provided increased ductility and, subsequently, the material’s ability to deform was remarkably increased.

### 3.5. Disintegration in Controlled Compost Soil

**Figure 8:**
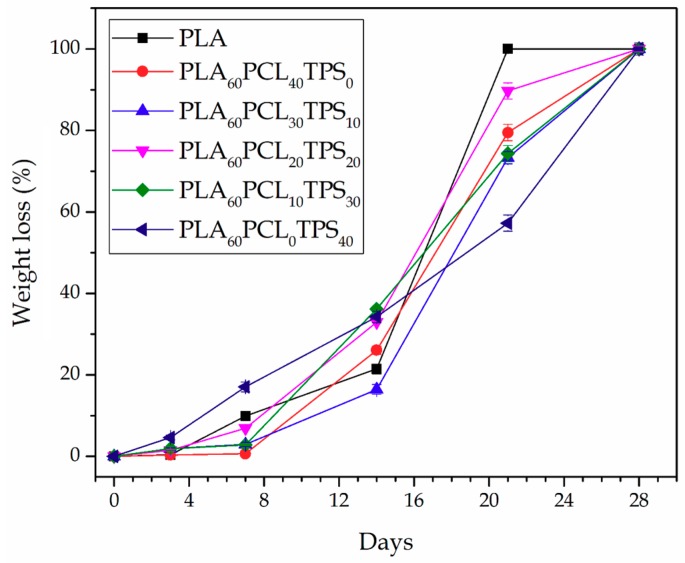
Evolution plot of the percentage of weight loss as a function of the elapsed time during disintegration in controlled compost soil of the polylactide (PLA), poly(ε-caprolactone) (PCL), and thermoplastic starch (TPS) blend pieces.

Figure 8 shows the percentage of weight loss as a function of the elapsed time during disintegration in the controlled compost soil of the injection-molded PLA-based pieces. One can observe that all of the here-prepared biopolymer pieces presented a significant loss of mass after only one week while they were fully disintegrated at the end of the test, that is, after a period of 28 days. The sample with the highest degradation rate was the neat PLA piece. In fact, after 21 days in the controlled compost soil, this sample already lost 100% of its initial weight. The addition of both PCL and TPS slightly reduced the biodegradation rate of PLA and this effect was more marked for the binary blend pieces, that is, PLA_60_PCL_40_TPS_0_ and PLA_60_PCL_0_TPS_40_, than for the ternary blend pieces. For instance, after 21 days, whereas the ternary PLA_60_PCL_20_TPS_20_ piece showed a mass loss of 89.9%, this value was only 57.3% for the binary PLA_60_PCL_0_TPS_40_ piece. This suggests that the biodegradation rate of PCL and TPS was lower than that observed for PLA in the selected compost soil. Therefore, the use of ternary blends improved the compostability profile of the binary blends made of PLA with PCL or TPS since, as shown during the morphological analysis, the regions of the secondary phases in the ternary blend pieces were smaller. Previous research studies have reported, however, that the PCL and TPS degradation rates are faster than that of PLA [67,68]. These differences can be ascribed to the type of culture present in the medium during disintegration. For instance, Thakore et al. [69] described that the different compost soils from municipal yard waste sites, which generally contains various types of microorganisms, can strongly affect the biodegradation profile of compostable biopolymer articles in a different manner. In particular, it was observed that the TPS degradation pathway was mainly produced due to two enzymes secreted by the microbes. In particular, esterase cleaves the ester bond, releasing free phthalic acid and starch, while amylase acts on starch to produce reducing sugars.

**Figure 9:**
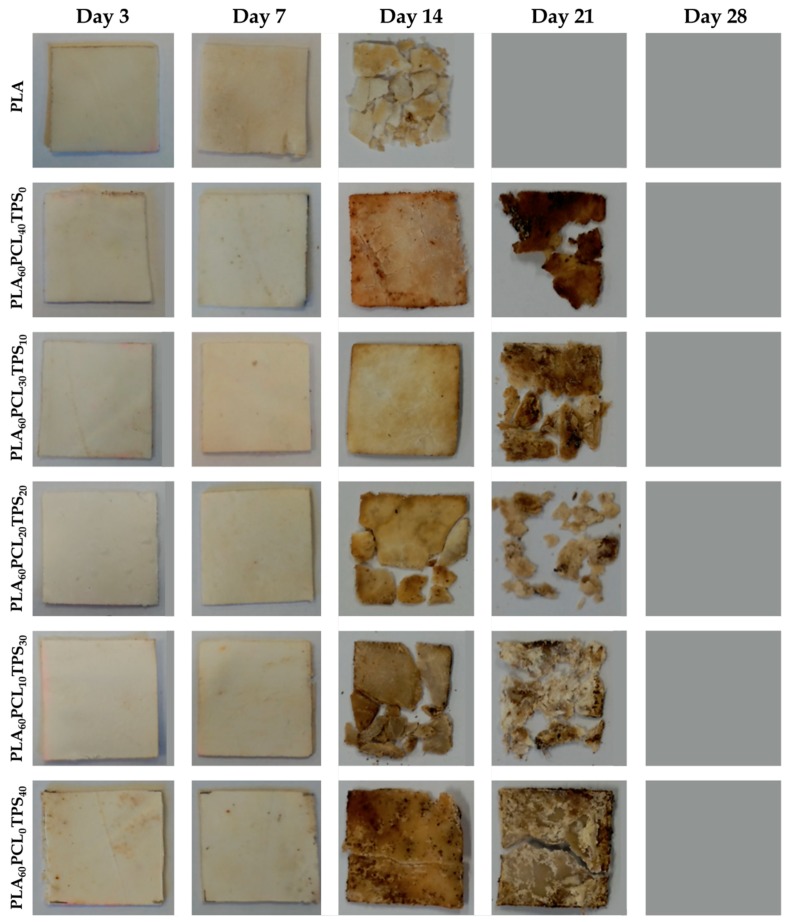
Visual aspect at selected disintegration times of the polylactide (PLA), poly(ε-caprolactone) (PCL), and thermoplastic starch (TPS) blend pieces.

Figure 9 finally shows the visual aspect of the injection-molded PLA-based pieces during the disintegration test, giving some further information about their compostability profile. After analyzing the samples appearance, one can conclude that all the PLA-based pieces were either fully disintegrated or significantly fragmented after 21 days. Regarding neat PLA, one can observe that its piece become opaque after only 3 days of incubation in the controlled compost soil due to hydrolysis of the biopolyester [70]. Although a slight weight decrease was observed, no significant alterations from a physical point of view (e.g., color changes, presence of micro-cracks, etc.) were seen during the first week. Over the second week, however, the PLA-based pieces revealed significant evidences of biodegradation. At this incubation time, the PLA piece as well as the PLA_60_PCL_20_TPS_20_ and PLA_60_PCL_10_TPS_30_ ternary blend pieces were extensively biodegraded producing small fragments. Although the other blend pieces, that is, the PLA_60_PCL_40_TPS_0_, PLA_60_PCL_30_TPS_10_, and PLA_60_PCL_0_TPS_40_, still remained into a single part, they visually presented a clear weight loss and develop a dark brown color. After 21 days, the neat PLA piece was fully biodegraded while the binary and ternary blend pieces were considerably disintegrated into small fragments, with the exemption of the binary blend piece of PLA with 40 wt % TPS, that is, PLA_60_PCL_0_TPS_40_. Therefore, as explained above, the addition of PCL and TPS slightly slowed down the disintegration process of PLA. This delay was mostly visible in the PLA-based pieces with high contents of either PCL or TPS, thought in the case of the plasticized carbohydrate it was even more pronounced. 

## 4. Discussion

Binary and ternary blend pieces based on PLA with different PCL and TPS contents are herein presented as novel sustainable plastics with improved ductility and toughness. In the here-performed tensile and flexural tests, it was observed that the addition of PCL and TPS provided a positive effect on flexibility and impact strength but also a slight reduction in the mechanical strength properties. Although both biopolymers individually produced a positive effect on the ductile properties of PLA, the best results were obtained for the ternary blends that combined high PCL contents with low amounts of TPS. In particular, the ternary blend piece of PLA with 30 wt % PCL and 10 wt % TPS, that is, PLA_60_PCL_30_TPS_10_, showed the highest flexibility with a ε_b_ value of 196.7%, approximately 40 times higher than that observed for the neat PLA piece. Similar findings were obtained in the impact tests, in which the ternary blends containing the highest PCL contents provided toughness increases of more than three times in comparison to the neat PLA piece. During the thermal analysis, DSC confirmed that the here-prepared binary and ternary blends are immiscible while TGA revealed that the ternary blend pieces present slightly higher thermal stability than the neat PLA piece and the binary blend pieces. Thermomechanical analysis, performed by means of DMTA, as well as VST and HDT measurements, also demonstrated that the blend pieces presented lower stiffness since both PCL and TPS effectively softened PLA. Finally, during the disintegration test in a controlled compost soil, it was observed that all PLA-based pieces presented a significant mass loss after only two weeks while the blend pieces disintegrated into small fragments after a period of 21 days. At the end of the test, that is, after 28 days, all pieces fully biodegraded. Although the addition of both PCL and TPS slightly reduced the PLA disintegration rate, this impairment was more marked for the binary blend pieces, that is, PLA_60_PCL_40_TPS_0_ and PLA_60_PCL_0_TPS_40_. Interestingly, the ternary blend pieces with intermediate contents of PCL and TPS presented a biodegradation rate close to that observed for the neat PLA piece.

## 5. Conclusions

The development of ternary blends based on PLA with relatively high contents of PCL and low contents TPS can be successfully applied for the development of compostable plastic articles with improved ductility and toughness. Potential uses of the here-described injection-molded pieces can be found in the rigid packaging industry, where for instance sustainable trays, bottles, and caps with high mechanical strength, but also sufficient ductility and impact strength, are currently required.

## Figures and Tables

**Table 1:** Composition and coding of the polylactide (PLA), poly(ε-caprolactone) (PCL), and thermoplastic starch (TPS) blends.

Sample	PLA (wt %)	PCL (wt %)	TPS (wt %)
PLA	100	0	0
PLA_60_PCL_40_TPS_0_	60	40	0
PLA_60_PCL_30_TPS_10_	60	30	10
PLA_60_PCL_20_TPS_20_	60	20	20
PLA_60_PCL_10_TPS_30_	60	10	30
PLA_60_PCL_0_TPS_40_	60	0	40

**Table 2:** Impact strength and Shore D hardness of the polylactide (PLA), poly(ε-caprolactone) (PCL), and thermoplastic starch (TPS) blend pieces.

Sample	Impact Strength (kJ·m^−2^)	Shore D Hardness
PLA	2.14 ± 0.28	73.1 ± 1.3
PLA_60_PCL_40_TPS_0_	6.52 ± 0.62	63.0 ± 1.0
PLA_60_PCL_30_TPS_10_	6.46 ± 0.39	63.6 ± 1.1
PLA_60_PCL_20_TPS_20_	6.51 ± 0.27	63.7 ± 1.2
PLA_60_PCL_10_TPS_30_	6.33 ± 0.24	64.3 ± 1.1
PLA_60_PCL_0_TPS_40_	5.46 ± 0.88	64.6 ± 1.1

**Table 3:** Values of the solubility parameters (δ) obtained for polylactide (PLA), poly(ε-caprolactone) (PCL), and thermoplastic starch (TPS).

Biopolymer	Chemical Structure	$\Sigma G\;(\mathrm{cal}/\mathrm{cc}){}^{1/2}$ [56]	δ (MPa^1/2^)
PLA	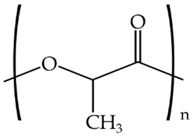	587	20.8
PCL	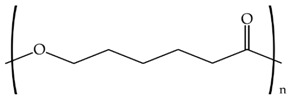	1010	19.4
TPS	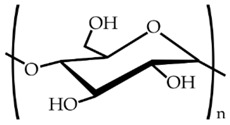	662	11.2

**Table 4:** Thermal degradation properties in terms of the onset degradation temperature (T_5%_), degradation temperature (T_deg_), and residual mass at 650 °C of the polylactide (PLA), poly(ε-caprolactone) (PCL), and thermoplastic starch (TPS) blend pieces.

Sample	T_5%_ (°C)	T_deg_ (°C)	Residual Mass (%)
PLA	322.67 ± 1.36	359.74 ± 1.58	1.5 ± 0.3
PLA_60_PCL_40_TPS_0_	325.03 ± 1.69	358.94 ± 2.14	0.4 ± 0.2
PLA_60_PCL_30_TPS_10_	303.50 ± 1.74	347.99 ± 2.36	3.2 ± 0.4
PLA_60_PCL_20_TPS_20_	315.33 ± 1.95	373.18 ± 1.74	1.2 ± 0.2
PLA_60_PCL_10_TPS_30_	332.06 ± 1.41	373.21 ± 1.95	5.7 ± 0.5
PLA_60_PCL_0_TPS_40_	320.34 ± 1.25	376.61 ± 1.78	6.4 ± 0.4

**Table 5:** Thermomechanical properties in terms of the Vicat softening temperature (VST) and heat deflection temperature (HDT) of the polylactide (PLA), poly(ε-caprolactone) (PCL), and thermoplastic starch (TPS) blend pieces.

Sample	VST (°C)	HDT (°C)
PLA	53.2 ± 0.5	47.9 ± 0.5
PLA_60_PCL_40_TPS_0_	51.2 ± 0.6	43.2 ± 0.4
PLA_60_PCL_30_TPS_10_	50.2 ± 0.5	46.4 ± 0.5
PLA_60_PCL_20_TPS_20_	48.8 ± 0.3	46.6 ± 0.4
PLA_60_PCL_10_TPS_30_	47.1 ± 0.5	46.2 ± 0.4
PLA_60_PCL_0_TPS_40_	47.4 ± 0.4	46.8 ± 0.3
